# Identification of Distant Drug Off-Targets by Direct Superposition of Binding Pocket Surfaces

**DOI:** 10.1371/journal.pone.0083533

**Published:** 2013-12-31

**Authors:** Marcel Schumann, Roger S. Armen

**Affiliations:** Department of Pharmaceutical Sciences, School of Pharmacy, Thomas Jefferson University, Philadelphia, Pennsylvania, United States of America; University of Copenhagen, Denmark

## Abstract

Correctly predicting off-targets for a given molecular structure, which would have the ability to bind a large range of ligands, is both particularly difficult and important if they share no significant sequence or fold similarity with the respective molecular target (“distant off-targets”). A novel approach for identification of off-targets by direct superposition of protein binding pocket surfaces is presented and applied to a set of well-studied and highly relevant drug targets, including representative kinases and nuclear hormone receptors. The entire Protein Data Bank is searched for similar binding pockets and convincing distant off-target candidates were identified that share no significant sequence or fold similarity with the respective target structure. These putative target off-target pairs are further supported by the existence of compounds that bind strongly to both with high topological similarity, and in some cases, literature examples of individual compounds that bind to both. Also, our results clearly show that it is possible for binding pockets to exhibit a striking surface similarity, while the respective off-target shares neither significant sequence nor significant fold similarity with the respective molecular target (“distant off-target”).

## Introduction

Searching for off-targets is very important for modern drug design and for ongoing efforts to understand the complex polypharmacology of well-known drugs. This search can be performed either in a ligand- or target-focused way. In the former case, the goal is to identify proteins to which an individual ligand might bind. Approaches to this include topology comparisons of ligands of different proteins and molecular receptor-ligand docking. In case of target-centered off-target searches, which will be the focus of this paper, the goal is to identify proteins (templates) whose ligand binding criteria are very similar to the ones of the molecular target of interest (query). Thus, many ligands of the query protein could be expected to also bind to the template protein.

Identification of off-targets in such a way would allow to potentially speed-up and rationalize drug design in several ways: drug targets that exhibit very many or very ‘dangerous’ off-targets (i.e. those potentially leading to severe medical problems in the patient), could be discarded as molecular targets. Alternatively, if a protein with identified off-targets is selected as molecular target, an emphasis can be placed in successive drug design steps to predict and verify the behavior of drug candidates on all those off-targets and inform the rational design of desired selectivity. In such way, side-effects of drugs could be prevented or detected early-on, long before entering clinical trails. Additionally, many rarer side-effects (that depend on a population subgroup or use of other drugs) might not even be encountered in clinical trials, but might in principle be detectable this way. Last but not least, off-target identification would also allow to better understand ligand selectivity relationships between proteins and the reason for side-effects of already commercially available drugs.

Several attempts have been made in the past to identify off-targets of a given protein target. A number of authors [1]–[5] have created fingerprints for description of the overall properties of a pocket and quick comparison of pockets. Spitzer *et al.* [6] have developed a procedure to compute surface similarities of superposed proteins. This approach does not focus on performing the actual superposition of the utilized proteins. As such, it can be used in combination with simple backbone-based superposition approaches or one of the procedures mentioned below in order to assess the binding pocket surface similarity of closely related proteins. In case of distant off-targets, it may on the other hand be desirable to directly and efficiently superpose the surfaces, which is the problem this paper focuses on. Similarily, Xie *et al.* [7] used a Gaussian density function on C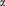 atoms to generate a statistical description of the similarity of the pockets of two given proteins, after employing a protein profile alignment based approach to superimpose these proteins. These authors have used this methodology for example to identify several nuclear hormone receptors as putative off-targets of CETP inhibitors [8]. However, due to the reliance of the method on sequence similarity, these off-targets still retain reasonable sequence similarity to the respective query protein.

Approaches performing an actual superposition of binding pockets onto each other commonly use a set of pseudo-centers, usually one per residue, to describe each pocket and then utilize a graph-matching algorithm to find a mapping of template protein pseudo-centers onto query pseudo-centers. The first such approach was described in Kuhn *et al.* [9], which was modified and extended in various way by several other authors [10]–[12]. However, all of those approaches display the same disadvantage if the goal is to identify distant off-targets: these approaches might suitably be described as local, pocket-centric fold comparison algorithms. They do not focus directly on the binding pocket or its surface, but use only a very rough representation in form of pseudo-centers. The influence of side chains to the shape of the binding pocket and to the ability of a ligand to bind to a protein, are furthermore commonly ignored. Comparisons of what kind of amino acids (or pseudo-centers) are found in what relative distance to each other might reasonably be expected to infer knowledge about local fold similarities of query and template proteins near the binding pocket and can thus be expected to be helpful to detect off-targets containing such a similarity to the query. While this is definitely very useful, an even harder goal is finding potential off-target that share neither significant sequence nor significant fold similarity with the target of interest. Using the aforementioned procedures towards this end would, for the described reason, most likely not be helpful.

Najmanovich *et al.* [13], on the other hand, developed an approach that uses graph-matching on all-atoms models of binding sites. Therefore, this procedure is not prone to the aforementioned problems acquired by a very rough description of the binding pocket. Nonetheless, this approach in a first steps explicitly searches the best superposition of template C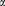 atoms onto query C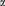 atoms, and only performs all-atoms graph matching on atoms that are near to each other as a result of the first step. Thus, this method effectually is a backbone matching procedure with subsequent minor, all-atom optimization. As such, it may be interesting for some cases; not so however if the goal is to find distant off-targets that share no fold similarity with the target structure.

An interesting approach by Hoffmann *et al.* [14] uses an all-atom model without utilizing a graph-matching. This procedure therefore does not depend on fold similarity and should be able to accurately model a pocket. However, the authors eliminate the need for a mapping of template onto query atoms (as performed by the aforementioned graph-matching algorithms) by simply calculating the similarity between two pockets as the sum over all distances between pairs of query and template atoms. This similarity is then optimized by use of a graph-based procedure. In order for a superposition of two pocket surfaces to be perfect, there is in general of course no need for this sum over all pairwise distances to be minimal. Thus, by use of this similarity for the application of off-target finding, the authors make the assumption that the entire template and query pockets are very similar with respect to shape, size and distribution of atoms. If some of these assumptions turn out to be false, as can reasonably be expected to be the case for the large majority of all possible pocket pairs, the approach, due to the utilized similarity function, has to end up simply placing the template pocket at the center of mass of the query pocket. This problem is furthermore aggravated by the fact that binding pockets can have significantly different (automatically determined) sizes and matching of sub-pockets is not possible this way.

Here, we present an approach to finding distant off-targets by direct superposition of protein binding pocket surfaces. We use a well-established ligand selectivity data set to show that our approach, although having being developed for detection of distant off-targets, can predict close off-targets as well as the state-of-the-art approach for that goal. We then apply our approach to a set of well-studied target proteins, searching the entire PDB for similar binding pockets, and for each of them thus reveal a convincing distant off-target candidate that shares no significant sequence or fold similarity with the respective target. Furthermore, the usefulness of these off-target definitions is confirmed by topology comparison of available, experimentally confirmed ligands for the respective target and off-target.

## Materials and Methods

Our approach superimposes two binding pockets, represented by atoms contributing to their SES (solvent-excluded surface), and afterwards scores the obtained superposition. We will first describe how we automatically determine the size of a binding pocket and will then explain how atoms are selected that should be used for the subsequent matching step. Afterwards, we will outline how the superposition is performed and how the final result is scored.

Note that if any water molecules are present near the ligand (within a convex hull of 5 Å) in the crystal structure, they will first of all be protonated, rotationally optimized and only water molecules that interact strongly with receptor and/or reference ligand are retained. For details about this, please see Schumann *et al.* [15].

### Determination of binding pocket size

Since the definition of the boundaries of the binding pocket has an impact on the following atom extraction step and since a binding pocket can be significantly larger than the area next to a specific reference ligand, we use an automatic procedure to determine the binding pocket size.

**Figure 1:**
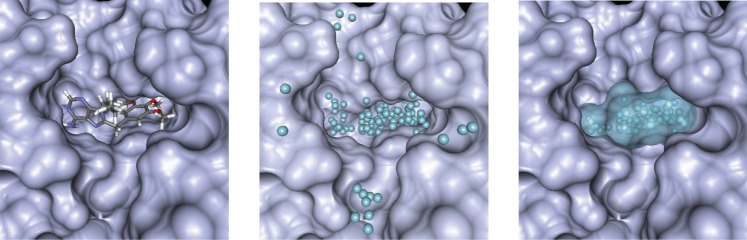
Pocket size detection. left) reference ligand in binding pocket; center) Place spheres in positions where they are relatively deeply burial in the receptor; right) Final pocket description, obtained by iteratively selecting spheres that are near to previously selected spheres, starting at geometrical center of ligand.

First, spheres are placed above the receptor surface in positions where they are deeply buried in the protein (i.e., in positions that are located above the SES surface and that have a high number of neighboring receptor atoms). We then sort these spheres according to their ascending distance to the geometrical center of the reference ligand. Starting with the sphere having the smallest distance, we add spheres to our pocket definition as long they have a distance smaller than 1.5 Å to at least one already selected sphere. Placing a bounding box around all spheres obtained this way reveals the size of the binding pocket. An example for the determination of the binding pocket size is shown in Fig. 1. Statistics about the size of the detected pockets of the query proteins for our distant off-target searches are shown in Table S1 in the Supplementary Material.

The spheres obtained by this procedure will also be used to define the solvent-exposed interior of binding pockets during the surface superposition step, as described below.

### Extraction of relevant surface atoms

In order to obtain the relevant binding pocket surface atoms, we first compute the SES for the given protein. Then, all protein atoms that do not contribute to the SES (according to a calculation with Conolly's analytical SES procedure [16]) are discarded. Afterwards, we check for each remaining atom whether its distance to the pocket bounding box (created as described above) or a bounding box placed around the reference ligand is smaller than 4 Å.

### Superposition of surfaces

In order to superpose template and query binding pocket surfaces, we developed a modification and extension of Katchalski-Katzir's [17] approach to protein-protein docking. In contrast to Kalchalski-Katzir, we will not aim to find the superposition resulting in the best docking pose of two proteins, but the one yielding the best binding pocket overlay.

First, we transform the set of atoms and spheres obtained in the previous steps into two separate three-dimensional grids (with a resolution of 1 Å) for query and template protein. Each cell of these grids will contain information, represented by numerical values, about whether its location is part of protein surface, directly (1 Å) above the surface, inside the binding pocket, or elsewhere on the outside of the protein. The grids thus contain a detailed representation of the three-dimensional shape and size of the binding pockets.

Each cell of the grid for the query protein is filled according to Eq. 1 in which 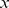, 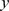 and 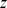 together denote a position in relative atomic units. Here, a grid cell is regarded as being part of the protein surface, if it has a distance smaller than 1.8 Å to any of the query protein atoms selected in the previous step. Similarly, a cell is defined as representing the interior of the binding pocket of the query protein if this cell has a distance smaller than 1.8 Å to any of the previously created spheres.
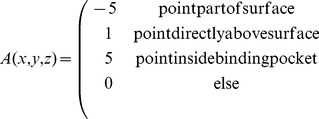
(1)


In a similar manner, a three-dimensional grid is filled for the template protein according to Eq. 2.
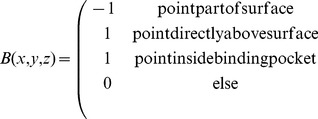
(2)


A correlation score describing the quality of a potential superposition obtained by transforming grid 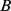 according to a given translation vector (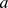, 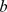, 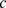) and a given rotation 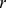, can thus be computed according to Eq. 3.
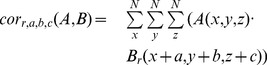
(3)


Values have been assigned to 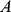 and 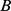 in the way shown in Equations 1 and 2 since they yield a positive correlation contribution (according to Eq. 3) in cases when either surfaces are matched onto each other or pocket areas are superimposed. Overlaying a surface with a pocket area, on the other hand, will be penalized this way, by means of a negative correlation contribution.

We then need to find the set of 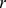, 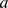, 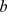 and 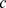 that yields the largest correlation value (Eq. 4). Naively, this could of course we achieved by iterating over all possible translations (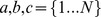) and rotation angles (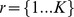). This would however require a total of approximately 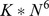 compute steps, so that the run-time using the naive implementation would in practice be much too high.
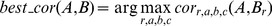
(4)


However, since a discrete Fourier transform (DFT) for a three-dimensional function has the form shown in Eq. 5, we can, as Katchalski-Katzir [17] discovered, utilize it to solve this kind of problem more efficiently.
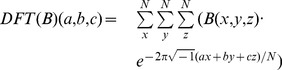
(5)


Thus, we can compute the correlation of 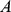 and 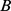, depended only on a given rotation, as
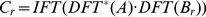
(6)


where IFT denotes the inverse Fourier transform and 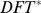 the conjugate of the DFT. Fourier and inverse Fourier transformations can be performed by the fast Fourier transform algorithm [18] (FFT), which requires only about 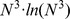 steps to transform our functions.

Thus, the best correlation can be found by only iterating over all rotations (instead of iterating over all translations and rotations, as shown in Eq. 4)
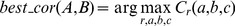
(7)


Values 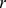, 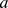, 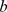 and 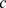 that yield this result are the optimal rotation and translation steps, respectively. In total, superposing the two grids this way hence requires only on the order of 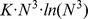 compute steps. For our evaluations, we discretize the rotations around the global X, Y and Z axis into steps of 20°. Run-time for superposing two binding pocket surface thus amounts to approximately ten seconds (on an AMD Opteron 6134).

### Scoring of superposition

To obtain a similarity score for two given binding pockets, we apply the best superposition found in the previous step and then compute the fraction of query surface that was matched to template surface and then we penalize for query pocket areas obscured by template protein:
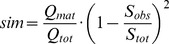
(8)


Here, 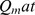 denotes the number of query surface atoms that have at least one template pocket surface atom within a distance of 1.8 Å and 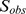 (obscured pocket spheres) denotes the number of query pocket spheres having at least one template surface atom within a distance of 1.4 Å. The total number of query surface atoms is represented by 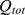, and the total number of query pocket spheres by 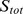.

### Availability

Created software tools are freely available as part of our computer-aided drug design suite (CADDSuite) at http://caddsuite.github.com.

## Results/Discussion

### Closely related off-targets

Although our approach is aimed at finding distant off-target that share no significant sequence or fold similarity with the respective target structure, we first of all evaluate our procedure on the data set utilized by Milletti [12] in order to show that we can indeed also adequately find relatively closely related kinase-kinase off-targets. This data set, a subset of the Ambit 2008 [19] panel, contains activity data for 17 compounds on 189 kinases, obtained by *in vitro* competition binding assays [19]. Given a co-crystal structure of a kinase with an inhibitor, the goal here is to predict which other kinases act as off-targets for the respective target structure.

**Figure 2:**
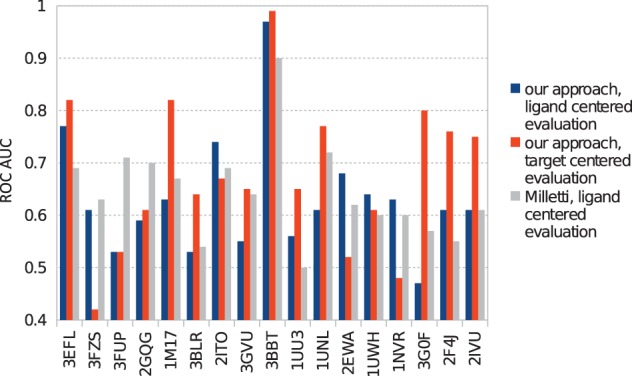
Evaluation of performance for identification of closely related off-targets of our approach (blue, orange) in comparison to Mellitti's [12] procedure (gray).

Comparison of the performance of our approach with the one by Milletti [12] as shown in Figure 2 in blue vs. gray, proves that we can predict close off-targets as well as the state-of-the-art approach for that goal. The average ROC AUC of Milletti over the 17 query pockets is 0.64, and our performance of 0.63 is not significantly different. (ROC curves for the individual targets are shown in Figure S1 in the Supplementary Material.)

However, is has to be noted that Milletti's “ligand centered” way of defining true off-targets may be considered suboptimal for evaluation of binding pocket comparisons. Milletti classifies a protein as a true off-target if the affinity of the target structure ligand to it was lower than 10 uM. The goal of pocket comparisons on the other hand is to find proteins that display a very similar binding pocket and might thus serve as off-target for a range of ligands, not just one particular ligand. As specificity of binding can vary considerably between different ligands, we perform a separate analysis of our results taking into account all ligands with available binding affinity data for target and off-target candidate. In our “target centered assessment”, we classify a protein as a true off-target, if it shares with the target protein at least half of their nano-molar inhibitors. Our average ROC AUC for all 17 query pockets, obtained by this target centered analysis, is 0.676. This shows that, even according to this more appropriate metric, our approach is able to find closely related off-targets well.

It is furthermore noteworthy that comparing results obtained by ligand vs. target centered analysis may be interesting in order to try to infer some knowledge about ligand selectivity and promiscuity. In eight out of 17 cases (1M17, 3BLR, 3GVU, 1UU3, 1UNL, 3G0F, 2F4J, 2IVU), the ROC AUC (receiver-operating characteristic area-under-the-curve) obtained by target centered analysis (shown in orange in Figure 2) was much larger than the one obtained with ligand centered assessment. This difference is most likely is due to strong and specific binding of the respective reference ligand to the target structure, whereas many other ligands bound less selectively. Therefore, the reference ligand, in contrast to other ligands, may not bind to most other examined proteins that do exhibit high binding pocket surface similarity. On the other hand, in three cases (3FZS, 2EWA, 1NVR) the quality was judged to be less good with the target centered analysis. Here, the explanation may lie in less selective binding of the reference ligand, compared to all other ligands of the respective target. This explanation can be easily rationalized by considering 1NVR, which is a co-crystal structure of staurosporine, the single most promiscuous kinase inhibitor. Thus, many ligands of the respective target may not bind to other examined proteins that show significant surface similarity, whereas the reference ligand was able to do so.

### Distant off-targets

In order to analyze the performance of our approach for identification of distant off-targets, we select a number of well-studied, medically relevant, kinase and non-kinase proteins as target structures and investigate whether we can find interesting and meaningful distant off-targets for them.

Therefore, binding pockets for all co-crystal structures in the PDB containing a ligand with a molecular weight between 300 and 800 g/mol are prepared in the aforementioned way. Each query pocket, after having being processed in a similar way, is then matched to each obtained PDB pocket (template) by our algorithm. Template proteins exhibiting a sequence identity greater than 40% or a secondary structure identity greater than 60% are ignored in these experiments in order to simplify the analysis of results since our goal is to find distant off-targets. Secondary structure identity is hereby calculated as the fraction of residues being part of an identical secondary structure type according to DSSP [20] after global alignment of query of template protein sequence. All superpositions generated for one query protein are subsequently ranked according to their pocket similarity scores as shown in Eq. 8. The approximately best ten matches are then analyzed manually in order to confirm surface similarity and fold dissimilarity.

For all examined query proteins, we could identify template proteins that share neither significant sequence nor fold similarity with the target but exhibit a high binding pocket surface similarity. In the following, we will discuss the results for all performed pocket searches. We will in each case describe the, in our opinion, medically most relevant off-target candidate. Each of them was encountered at the very top of the rank list (within the approximately top 10). To further strengthen the suggested connection between target and off-target, we will then compare experimentally confirmed strong binders (affinity lesser than 5 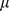
M) of the target with those of the off-target using data available in BindingDB [21]. The basic intent of these searches for similar binding pockets is, as mentioned before, to find proteins that could realistically acts as off-target for a range of ligands of a given molecular target, not to predict the binding or selectivity of any individual ligand.

#### CLK3

The first search for potential distant off-targets is performed for CDC-like kinase 3 (CLK3). CLK3 acts as a dual specificity, serine/threonine and tyrosine kinase that phosphorylates serine- and arginine-rich splicing factors and is thus assumed to be involved in the regulation of mRNA splicing and alternative splicing [22], [23].

**Figure 3:**
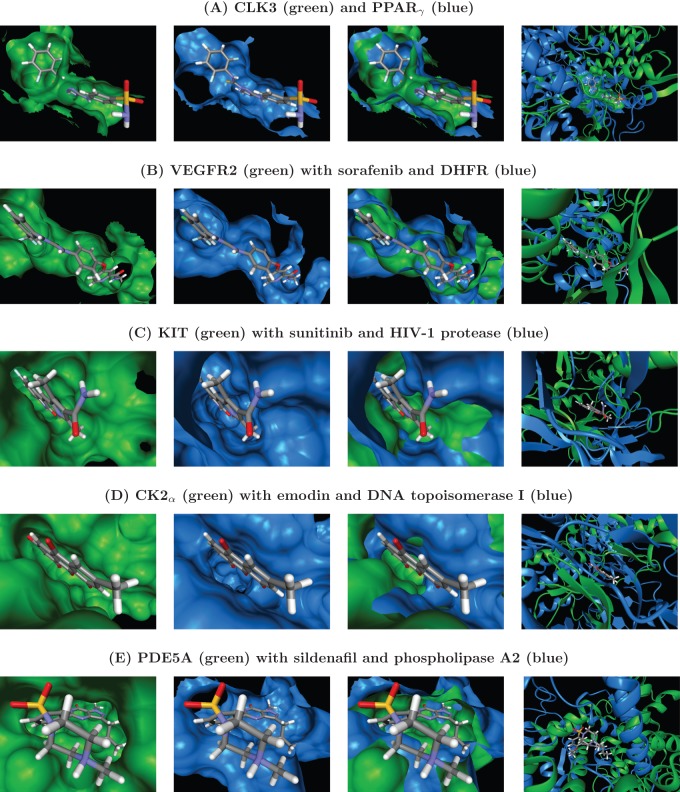
Identification of distant off-target candidates by search of PDB pockets. The query protein is shown in green; the template protein in blue. Columns 1–3 show the binding pocket surfaces of query, template, template and query, respectively, together with the query structure's reference ligand. Were necessary, surfaces have been cut open for better view. Column 4 depicts the fold dissimilarity near the binding pocket. Part 1 of 2.

**Figure 4:**
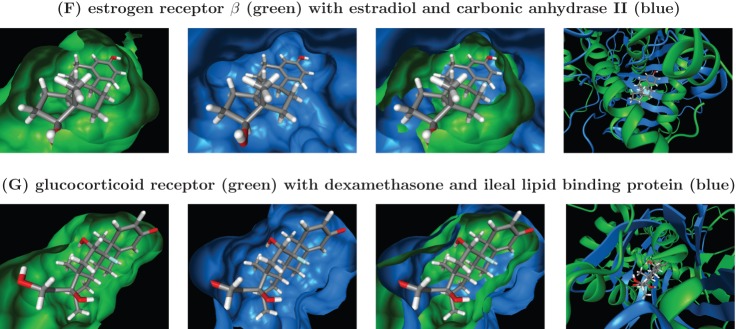
Identification of distant off-target candidates by search of PDB pockets. Part 2 of 2.

**Figure 5:**
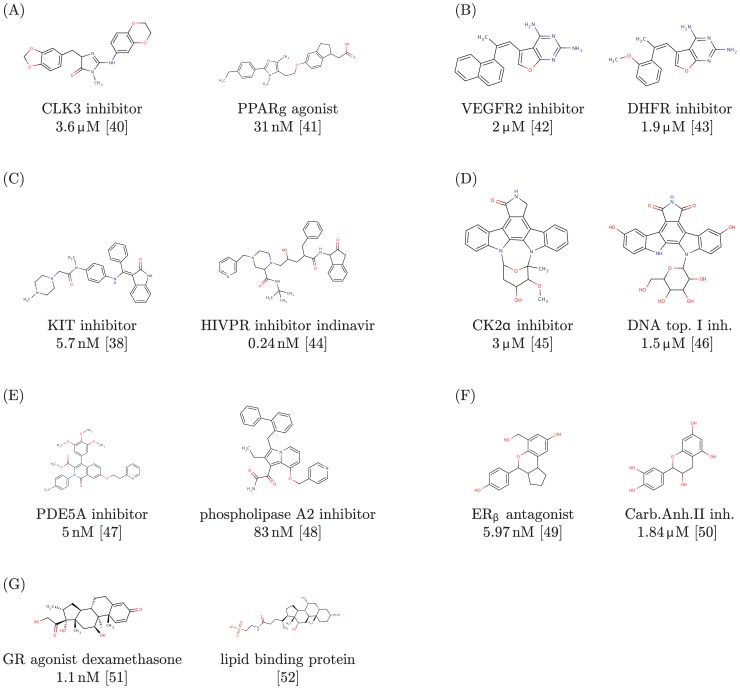
Known ligands for each of the query proteins, together with known ligands of each suggested off-target.

Analysis of the results obtained for CLK3 (PDB ID 2WU6) shows a striking similarity, shown in Figure 2a, of its binding pocket to the one of peroxisome proliferator-activated receptor gamma (PPAR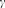, PDB ID 3R8I). PPAR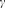 is a nuclear receptor that activates the acyl-CoA oxidase transcription and thereby has a important impact on fatty acid metabolism [24]. CLK3 and PPAR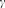 show a very low sequence identity of only 23% but their binding pockets exhibit a very similar shape and size. Furthermore, the folds of the two proteins near the superposed binding pockets are completely dissimilar, as can be clearly seen in Figure 3a (See also Figure 4). Thus, PPAR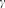 may be considered a distant off-target candidate for CLK3. Comparison of known strong binders for CLK3 with those for PPAR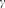 reveals compounds, shown in Figure 5a, with a very high topological similarity. Furthermore, this pair of ligands is also very similar to reference ligand in the utilized co-crystal for CLK3. Taken together, these findings indicate that it may be very helpful to consider PPAR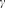 as an off-target when trying to develop drugs for CLK3 (or vice versa) and thus try to predict and/or experimentally measure the affinity of potential drug candidates to both proteins.

Furthermore, in other pocket searches, we have observed numerous examples of similarity between other human kinase enzyme pockets (that are themselves similar to our CLK3 query protein 2WU6) and PPAR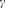. Thus, we hypothesize that various classes of kinase inhibitor scaffolds may have the potential to exhibit PPAR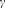 partial agonist activity. One literature example of a drug candidate molecule that displays this behavior is ertiprotafib, which was initially developed as a PTP1B inhibitor, and later found to act as partial agonist of PPAR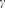 [25], [26] and potent inhibitor of IkappaB kinase beta (IKK-beta) [27].

Observations regarding other drug classes also help to substantiate the hypothesis that the binding pocket of PPAR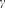 exhibits remarkable flexibility resulting in high pocket similarities to a significant number of other unrelated proteins. The sulfonylurea class of commonly prescribed antidiebetic agents (glipizide, glyburide, glimepride) primarily target the sulfonylurea receptor stimulating insulin release, where the thiazolidinedione class of antidiebetic agents (pioglitazone) primarily act as partial agonists of PPAR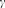 improving insulin resistance through the transcription of the insulin-sensitive genes involved in the control of glucose and lipid metabolism. A very interesting paper by Scarsi *et al.* [28], demonstrated convincing evidence that glipizide (the most commonly prescribed of the sulfonylurea class) exhibits PPAR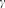 partial agonist activity as well as having primary activity at the sulfonylurea receptor, thus showing Glipizide's theraputic efficacy may have origins in dual activity for both drug targets. Interestingly, there is recent evidence from the literature for biologically relevant PPAR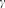 partial agonist off-target activity for examples from three other different major drug target classes. Partial agonist activity for PPAR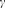 has recently been reported for an angiotensin receptor antagonists (telmisartan) [29], the most commonly used ACE inhibitor (lisinopril) [30, 31], and in the COX 1/2 inhibitor indomethacin [32]. The observation that drugs from four different classes (glipizide, telmisartan, lisinopril, and indomethacin) all exhibit partial agonist activity for PPAR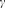, support our observation that agonist-bound conformations of the PPAR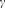 pocket in particular may exhibit similarity to other drug target pockets.

#### VEGFR2

Vascular endothelial growth factor receptor 2 (VEGFR2) is a tyrosine protein kinase that serves as a receptor for growth factors and thus has a strong influence on endothelial cell growth, migration and differentiation [33].

The VEGFR2 binding pocket (as observed in PDB entry 4ASD) is revealed by our approach to have a very high similarity, shown in Figure 3b, to the one of dihydrofolate reductase (DHFR, PDB ID 3SA1). DHFR converts dihydrofolic acid to tetrahydrofolic acid, a key step in the folic acid pathway that is necessary for generation of precursors of DNA and glycine [34]. Sequence identity between the utilized VEGFR2 and DHFR structures is just 22% and Figure 3b furthermore reveals that the two proteins share no fold similarity that could serve as a trivial explanation for the high observed pocket similarity. Although it is not the intent of our procedure to predict the activity of individual compounds, it is noteworthy that the VEGFR2 reference ligand, sorafenib, seems in principle to be well accommodated by the DHFR pocket. Even if sorafenib itself turned out not bind to DHFR *in vivo*, this still visualizes that DHFR might act as off-target for other compounds (related to sorafenib) that act on VEGFR2 (or vice versa). This connection between VEGFR2 and DHFR is also strengthened by the fact that some very similar ligands, depicted in Figure 5b, have already been proven to bind to either of them with similar affinities (2 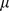
M and 1.9 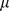
M, respectively). A superposition of the DHFR pocket that was found in the search (PDB ID 3SA1) with the E and Z derivatives of the compound shown in Figure 5b (PDB ID 3K45 and 3K47) [35] bound to human DHFR shows that the binding modes of all three DHFR inhibitors are quite similar within the pocket that was matched in the search. The authors of the crystal structures [35] are rationally developing these compounds as dual inhibitors of tyrosine kinases and DHFR.

#### KIT

KIT is a tyrosine protein kinase acting as a receptor for cytokines that is involved, via various pathways, in (among others) cell survival, migration, differentiation and melanogenesis [36].

A strong binding pocket similarity is detected utilizing our approach between KIT (PDB ID 3G0E) and HIV-1 protease (HIVPR, PDB ID 3NLS), as can be seen in Figure 3c. HIVPR cleaves polyproteins of human immunodeficiency virus (HIV) into separate proteins that then make up the viral envelope (virion) of HIV and is thus necessary for HIV infectivity [37]. The KIT and HIVPR structures exhibit a sequence identity of only 21% and no fold similarity (see Figure 3c). The observed pocket of HIVPR is slightly shorter than the KIT pocket in the direction of the fluorobenzene group of sunitinib shown in the superpositions, but otherwise displays very high similarity. Thus, it might be desirable to keep KIT in mind as potential off-target when trying to develop drug for HIVPR. Figure 5c furthermore shows that indeed a compound that is very similar to the HIVPR inhibitor indinavir has already been proven to strongly bind to KIT. The affinities of both molecules are in the low nano-molar range (0.24 nM in case of indinavir [38] and 5.7 nM [38] in case of the similar KIT inhibitor). In addition, a very interesting paper by Xie *et al.* [39], has recently computationally identified and then experimentally verified that the HIVPR inhibitor nelfinavir indeed demonstrates the ability to inhibit multiple kinases.

#### CK2 *α*

Casein kinase II subunit alpha (CK2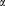) is the catalytic subunit of casein kinase II, a serine/threonine protein kinase complex involved in many signaling cascades, including some affecting cell cycle progression [53] and apoptosis [54].

Application of our pocket comparison approach reveals, as shown in Figure 3d, that the pocket of DNA topoisomerase I (as encountered in PDB entry 1SEU) has a highly similar shape and size, compared to the CK2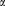 binding pocket (PDB ID 3Q9X). DNA topoisomerase I creates single-strand cuts of DNA and subsequent reconnection after release of DNA supercoiling. Thus, DNA topoisomerase I is important for DNA replication. CK2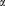 and DNA topoisomerase I share a sequence identity of only 21% and no fold similarity, as depicted in Figure 3d, that could explain the highly similar pockets. Examination of known CK2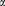 and DNA topoisomerase I binders furthermore revealed very similar compounds (see Figure 5d), both of which are related to staurosporine, that have been shown to bind with similar affinities to either of the two proteins. The superposition of the human topoisomerase I structure bound to the staurosporine related indocarbazole shown in Figure 5d (PDB ID 1SEU) [55], with the structure of the FDA approved drug hycamptin (PDB ID 1K4T), reveals that the pocket match is to the biologically relevant binding mode of the camptothecin class of topoisomerase I poisons [55].

#### PDE5A

Phosphodiesterase 5A (PDE5A) is a phosphodiesterase that converts cGMP into GMP and is thereby (among others) involved in the relaxation of smooth muscles [56].

A very high pocket similarity, displayed in Figure 3e, is revealed by our approach between the sildenafil-bound PDE5A pocket (PDB ID 1TBF) and the binding pocket of phospholipase A2 (PLA2, PDB ID 1FXF). Phospholipase A2 hydrolyzes the sn-2 acyl bond of arachidonyl phospholipids, releasing arachidonic acid. Its function is implicated in the initiation of the inflammatory response and thus has been the target of drug discovery efforts [57] for anti-inflammatory agents, especially for neurological [58] and cardiovascular [59], [60] indications. PDE5A and PLA2 contain only 20% sequence identity have no fold similarity near the superimposed pockets, as can be seen in Figure 3e. Also, molecules with significant topological similarity to each other and to sildenafil are known to bind strongly to PDE5A, respectively PLA2 (see Figure 5e).

Phospholipase A2 inhibition has also been identified as off-target activity in some commonly used drugs. Non-pancreatic secretory phospholipase A2 (membrane associated) is inhibited by diclofenac [61], [62], and cytosolic phospholipase A2 is inhibited by the commonly prescribed corticosteroid fluticasone propionate [63], [64], as well as epirubicin [65] and niflumic acid [66].

#### Estrogen receptor *β*

Estrogen receptor 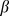 (ERb) is a nuclear receptor that activates the transcription of genes containing estrogen response elements. Its estradiol-bound pocket (PDB ID 3OLL) is shown by our pocket comparison to be highly similar (see Figure 4f) to the pocket of carbonic anhydrase II (CAII, PDB ID 3OKV). CAII is no nuclear steroid receptor but the ubiquitous enzyme that catalyzes the conversion carbon dioxide to carbonic acid. As such, it shares only a sequence identity of 24% with ERb. Furthermore, the high pocket similarity is not the result of any significant fold similarity, as is clearly visualized in Figure 4f.

**Figure 6:**
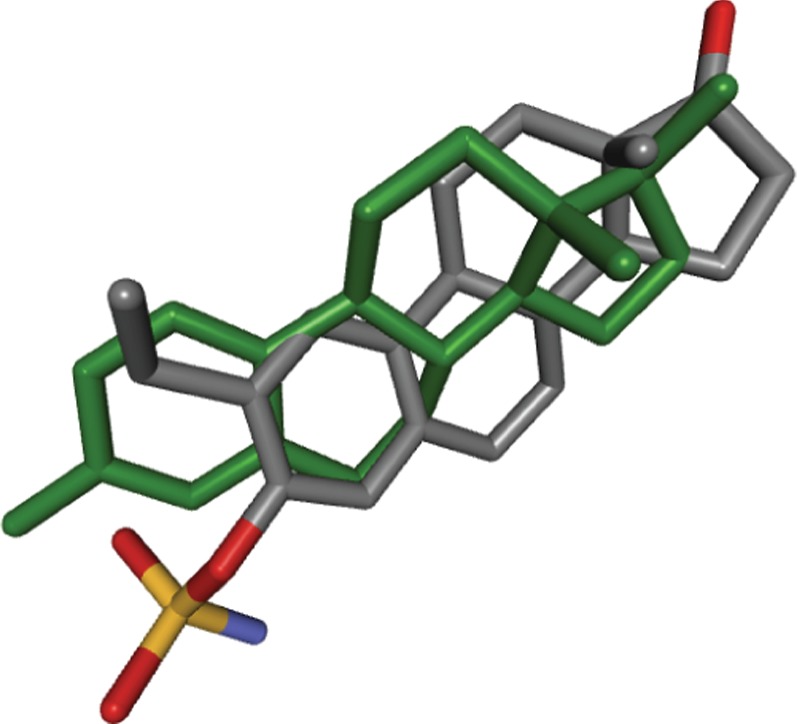
Crystal structure pose of estradiol in 3OLL (green) in comparison to the pose of the ligand of 3OKV obtained by superposing the latter onto the former by use of our algorithm.

In contrast to all results discussed before, the ligand observed in the co-crystal structure of the target and the one in the structure of the suggested off-target have a very similar topology. Both compounds are derivatives of estrogen. Therefore it is of interest that the obtained pocket superpositions resulted in very close poses for two ligands, shown in Figure 6. In cases in which reference ligands of target and suggested off-target are highly similar, a simply querying of the PDB for a given ligand topology could arguably have yielded similar results. However, doing so would not elucidate whether binding of similar ligands by target and off-target is due to ligand promiscuity or binding pocket similarity. Since we, after having used our pocket superposition based search, now know that the pockets of ERb and CAII can assume very similar shapes, we searched their respective BindingDB [21] data sets for other known topologically similar ligands. We indeed found strong inhibitors of CAII that are highly similar to strong antagonists of ERb and that are not steroids, displayed in Figure 5f.

#### Glucocorticoid receptor

Glucocorticoid receptor (GR) is a nuclear receptor regulating the transcription of genes containing glucocorticoid response elements that is thereby involved in modulating inflammatory response and other processes.

**Figure 7:**
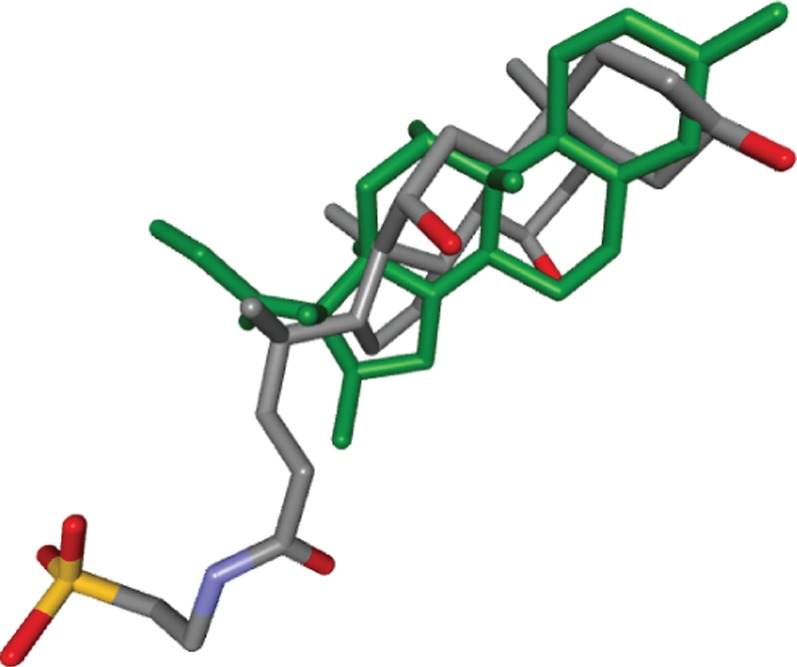
Dexamethasone as observed in 3MNP (green) in comparison to taurocholic acid in 1O1V after superposition of the two pockets by our approach.

As can be seen from Figure 4g, the dexamethasone-bound binding pocket of GR (PDB ID 3MNP) was found by our pocket comparison to be highly similar to the pocket of ileal lipid binding protein (ILBP, PDB ID 1O1V). ILBP is involved in the transportation of bile acids. While bile acids as well as glucocorticoids are steroids and thus this pocket search result may come at no surprise, this example visualizes that our pocket matching approach correctly superposes binding pockets that are known to bind near identical ligands. The very low deviation between the pose of the ILBP ligand and the GR-bound dexamethasone pose, obtained after applying our pocket superposition, is shown in Figure 7. Although the ligands of these two proteins are, as stated, very similar, the pocket surface superposition was not trivial, since both proteins have a sequence identity of only 23% and, as Figure 3g makes clear, share no fold similarity that might have produced the high pocket similarity.

#### Comparison to other methods

As described above, and as can be seen in Figures 3–4, all of our distant off-target matches exhibit no significant backbone and no significant fold superposition. Thus, the use of an accurate description of the binding pocket was important here for the superposition, so that simpler approaches, for example, C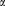 atom-based procedures, would not have sufficed to find these off-targets. To furthermore substantiate this, we now perfomed all pocket superpositions between target and repective proposed distant off-target using only C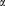 atoms. Thus, all previously utilized residues are now used as well, but instead of using all atoms that contribute to the pocket surface (and pocket spheres), we now used only those residues' C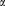 atoms. Afterward performing the superpositions, they are scored in the normal way, according to Eq. 8. The average pocket superposition score when using only C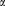 atoms for the matching, was 0.095, compared to 0.614 when using the normal superposition approach. (Scores for individual superpositions are shown in Table S2 in the Supplementary Material.) Visual examination furthermore showed that when using only C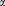 atoms for the superposition, the interior of the superposed off-target protein obscured large parts of the target's pocket (or vice versa), rendering these C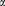 atom-based superpositions practically useless. This confirms that inclusion of an accurate pocket (surface) description, utilizing all relevant atoms (including side chain atoms), was crucial for identification of the off-targets proposed here.

Additionally, we checked whether existing approaches would have found any of the presented distant off-targets. Note however, that existing approaches cannot be reasonably excepted to discover distant off-targets, since they were not designed for this and since they, in constrast to our method, rely on sequence- or fold similarity, as explained in the introduction. First, we used PoSSuM [5] (http://possum.cbrc.jp/PoSSuM/) without a limit for the maximum number of hits to be reported, and searched the entire PDB database for protein pockets similar to each of our seven query proteins. For all seven cases, PoSSuM did not find the off-target of the respective query protein. Next, we utilized ProBis [11] (thttp://probis.cmm.ki.si) to individually compare each of our query pockets to its respective presented off-target pocket. For six out of seven cases, ProBis detected no similarity at all. In the case of CK2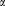, ProBis found only a very low pocket similarity to DNA topoisomerase I (z-score of 1.08). The three-dimensional superposition of these two proteins generated by ProBis furthermore mapped the CK2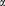 pocket onto the interior of DNA topoisomerase I, showing that no meaningful pocket similarity could be detected.

## Conclusions

In this paper, we presented a new approach for identification of distant drug off-targets. Protein atoms near the binding pocket surface and information about the location of the interior of the binding pocket are converted into numerical values and stored in a three-dimensional grid. Performing this step separately for query and template protein results in two grids, a multiplication of which yields a score for a potential superposition of the pockets of the two proteins. Fast Fourier transformation is then utilized to speed-up the search for the best superposition by about three orders of magnitude compared to a naive implementation and thus allows for fast and efficient comparison of binding pockets.

We demonstrated that our approach is able find convincing distant off-target candidates that share no significant sequence or fold similarity with the respective molecular target. The connection between targets and suggested off-targets was additionally strengthened by the high topological similarity between some known strong binders of the target and the respective off-target, and by literature examples of ligands that exhibit experimentally confirmed activity to both respective proteins.

Thus, applying our approach in order to derive a list of off-target candidates to be taken into account if trying to develop new drugs may be very helpful. By doing so, the affinity of each drug candidate to the suggested off-target can then be predicted or experimentally measured. Drug candidates that are detected to strongly interact with off-targets can hence be cast aside, potentially speeding-up the process of development of new drugs and helping to evade side-effects and toxicity. Furthermore, significant flexibility of the query protein is not by itself problematic due to two important methodological reasons. First, our approach will always determine the best superposition of two pockets, even if this results in only parts of the two pockets being matched onto each other. Secondly, since we always search the entire PDB database for similar template pockets, the search itself allows for significant flexibility between different templates. However, problems can arise if the template protein belongs to a class of proteins for which there are only very few structures in the PDB data base (e.g., transmembrane proteins). If it is suspected that a given protein has (distant) off-targets that belong to such a class, it might be of interest to perform the search as described in this manuscript step-by-step for different parts of the pocket. A variety of different techniques (e.g., elastic network models, molecular dynamics) could be used to assess the flexibility of different parts of the query pocket. For example, a search could thus begin with the most static query pocket region, and subsequent searches could be performed using diverse conformational states of other parts of the query pocket.

Although the presented approach, in its current form, is aimed at finding off-targets that could potentially bind a large range of target ligands and not to study individual compounds, future combinations of this approach with successive molecular docking, scoring and/or ligand pose optimizations (e.g. energy minimizations) could allow to also investigate the potential behavior of individual ligands in more detail.

## Supporting Information

Figure S1 **ROC curves showing the performance of our approach on identification of close off-targets for all 17 of Milletti's** [**12**] **data sets.** Results for target-centered evaluation are shown in blue, results for ligand-centered evaluation in orange (see text for explanation).
(PDF)

Table S1 **Size and number of residues contributing to pocket surface for all query proteins used for our distant off-target searches.**
(PDF)

Table S2 **Scores for distant off-target pocket superpositions performed by our approach in the normal way, in comparison to when using only C_α_ atoms for the superpositions.** Superposition scores were calculated as shown in Eq. 8.
(PDF)
