# Supplementary figures and images for: Identification of Distant Drug Off-Targets by Direct Superposition of Binding Pocket Surfaces
Source: PLoS One. 2013 Dec 31;8(12):e83533. doi: 10.1371/journal.pone.0083533 (PMC3877058; doi:10.1371/journal.pone.0083533)

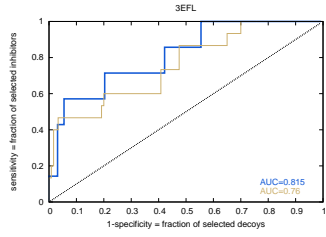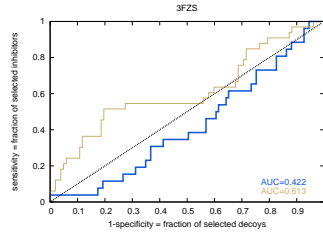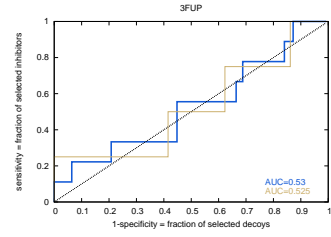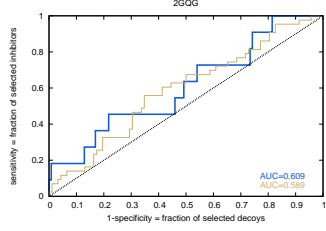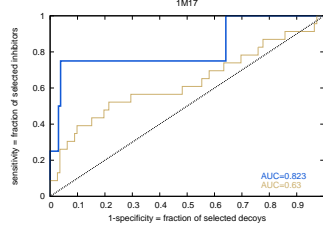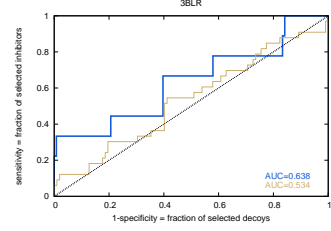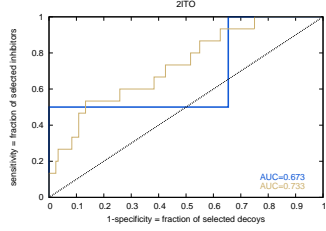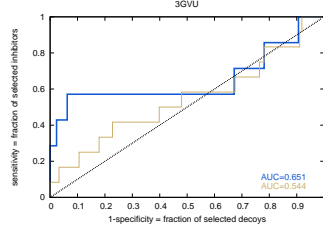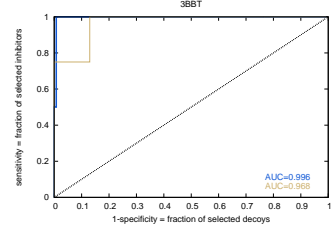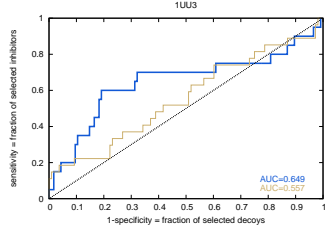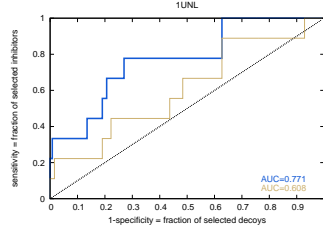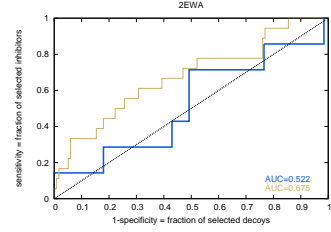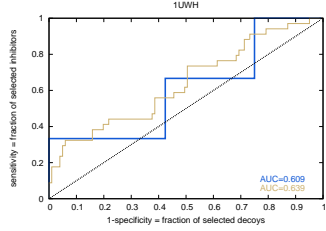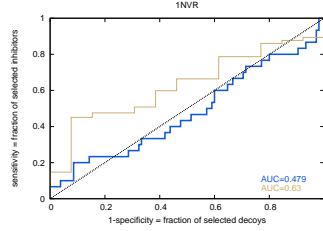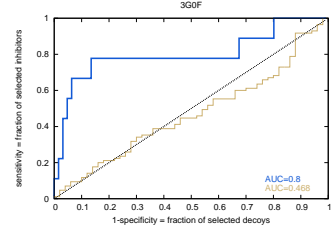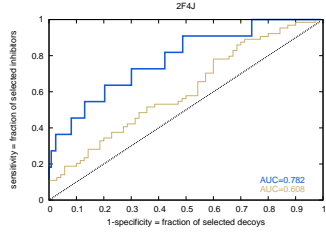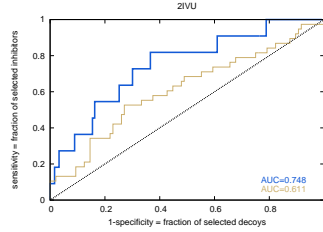

Supplement: Figure S1 — ROC curves showing the performance of our approach on identification of close off-targets for all 17 of Milletti's [12] data sets. Results for target-centered evaluation are shown in blue, results for ligand-centered evaluation in orange (see text for explanation). (PDF) [file pone.0083533.s001.pdf]
