# Supplementary material for: Identification of Distant Drug Off-Targets by Direct Superposition of Binding Pocket Surfaces
Source: PLoS One. 2013 Dec 31;8(12):e83533. doi: 10.1371/journal.pone.0083533 (PMC3877058; doi:10.1371/journal.pone.0083533)
Supplement: Table S1 — Size and number of residues contributing to pocket surface for all query proteins used for our distant off-target searches. (PDF) [file pone.0083533.s002.pdf]

query protein	pocket size	#residues
2WU6	13.5 x 9 x 7 Å	49
4ASD	13 x 7.3 x 7.6 Å	54
3G0E	11.2 x 9 x 5 Å	32
3Q9X	12.1 x 10.7 x 13.8 Å	44
1TBF	10 x 8.5 x 3.5 Å	50
3OLL	9.4 x 7.4 x 5.7 Å	31
3MNP	10.1 x 8.25 x 6.4 Å	35
