# Supplementary material for: Identification of Distant Drug Off-Targets by Direct Superposition of Binding Pocket Surfaces
Source: PLoS One. 2013 Dec 31;8(12):e83533. doi: 10.1371/journal.pone.0083533 (PMC3877058; doi:10.1371/journal.pone.0083533)
Supplement: Table S2 — Scores for distant off-target pocket superpositions performed by our approach in the normal way, in comparison to when using only Cα atoms for the superpositions. Superposition scores were calculated as shown in Eq. 8. (PDF) [file pone.0083533.s003.pdf]

	score	
protein pair	C_α only	normal
2WU6 - 3R8I	0.025	0.66
4ASD - 3SA1	0.09	0.6
3G0E - 3NLS	0.19	0.49
3Q9X - 1SEU	0.025	0.58
1TBF - 1FXF	0.2	0.51
3OLL - 3OKV	0.13	0.71
3MNP - 1O1V	0	0.7
average	0.095	0.614
